# The origin and population genetic structure of the ‘golden tide’ seaweeds, *Sargassum horneri*, in Korean waters

**DOI:** 10.1038/s41598-019-44170-x

**Published:** 2019-05-23

**Authors:** Seo Yeon Byeon, Hyun-Ju Oh, Sangil Kim, Suk Hyun Yun, Ji Hyoun Kang, Sang Rul Park, Hyuk Je Lee

**Affiliations:** 1 Molecular Ecology and Evolution Laboratory, Department of Biological Science, College of Science and Engineering, Sangji University, 26339 Wonju, Republic of Korea; 2 Oceanic Climate and Ecology Research Division, National Institute of Fisheries Science, 46083 Busan, Republic of Korea; 3 Korean Entomological Institute, Korea University, 02841 Seoul, Republic of Korea; 4 Estuarine and Coastal Ecology Laboratory, Department of Marine Life Sciences, Jeju National University, 63243 Jeju, Republic of Korea

**Keywords:** Molecular ecology, Population genetics

## Abstract

In recent years, drifting and inundating brown seaweed (*Sargassum horneri*) biomass, called ‘golden tides’, has frequently drifted and accumulated along the southern coastlines of Korea, causing devastating impacts on the local economy and coastal ecosystems. In this study, based on combined analyses of mitochondrial DNA *cox3* gene and seven microsatellites, we investigated the genetic makeup of the floating *S*. *horneri* populations (*N* = 14) in comparison to Korean benthic populations (*N* = 5), and tracked their genetic sources. Given a shared mtDNA haplotype and oceanic circulation systems, the floating populations may have been originated from the southeastern coast of China (e.g. Zhoushan, Zhejiang province). Population structure analyses with microsatellites revealed two distinct genetic clusters, each comprising floating and benthic populations. High levels of inter-population differentiation were detected within Korean benthic samples. The floating populations from the same periods during a 2015–2018 year were genetically more different from one another than those from different periods. These results suggest that the floating populations might be of multiple genetic sources within geographic origin(s). This study will inform management efforts including the development of “*S*. *horneri* blooming forecasting system”, which will assist in mitigating ecological and economic damages on the Korean coastal ecosystems in the future.

## Introduction

Recurrent outbreaks of drifting seaweed masses, called ‘seaweed tides’, are a nuisance phenomenon worldwide, as they pose a serious threat to local biodiversity in coastal ecosystems and also cause severe damage to the local economy by disturbing tourism, aquaculture and fisheries^1–3^. The green seaweeds, *Ulva* including formerly known as genus *Enteromorpha* and the brown macroalgae, the genus *Sargassum* are especially notorious for the formation and inundation of drifting seaweed biomass along the coastlines globally^2^. Shortly after being accumulated along the coastlines forming like carpets, these seaweeds begin stinking by releasing toxic hydrogen sulphide (H_2_S), rendering devastating effects on the coastal ecosystems^2,4^. The physiologically atypical capability of sustaining their lives as an unattached (drifting) form of these algae enables them to easily build up a large amount of biomass, colonize new space, and form the massive seaweed tides^1^.

Drifting biomass of *Sargassum* spp., called “golden tides”^2^ accumulates nearshore coastal waters usually during spring to summer, which causes detrimental effects on aquaculture and fisheries and also the whole coastal ecosystems in many parts of the world including the Gulf of Mexico, Caribbean, West Africa, eastern Pacific coast, China coast and the southwestern coast of Korea^5–9^. This phenomenon of the golden tides has been intensified dramatically in frequency, range, and magnitude over recent years perhaps due to growing anthropogenic pressure, such as climate change, nutrient loadings (e.g. coastal eutrophication) and inadvertent algal species transport by ship movements^10,11^.

**Figure 1:**
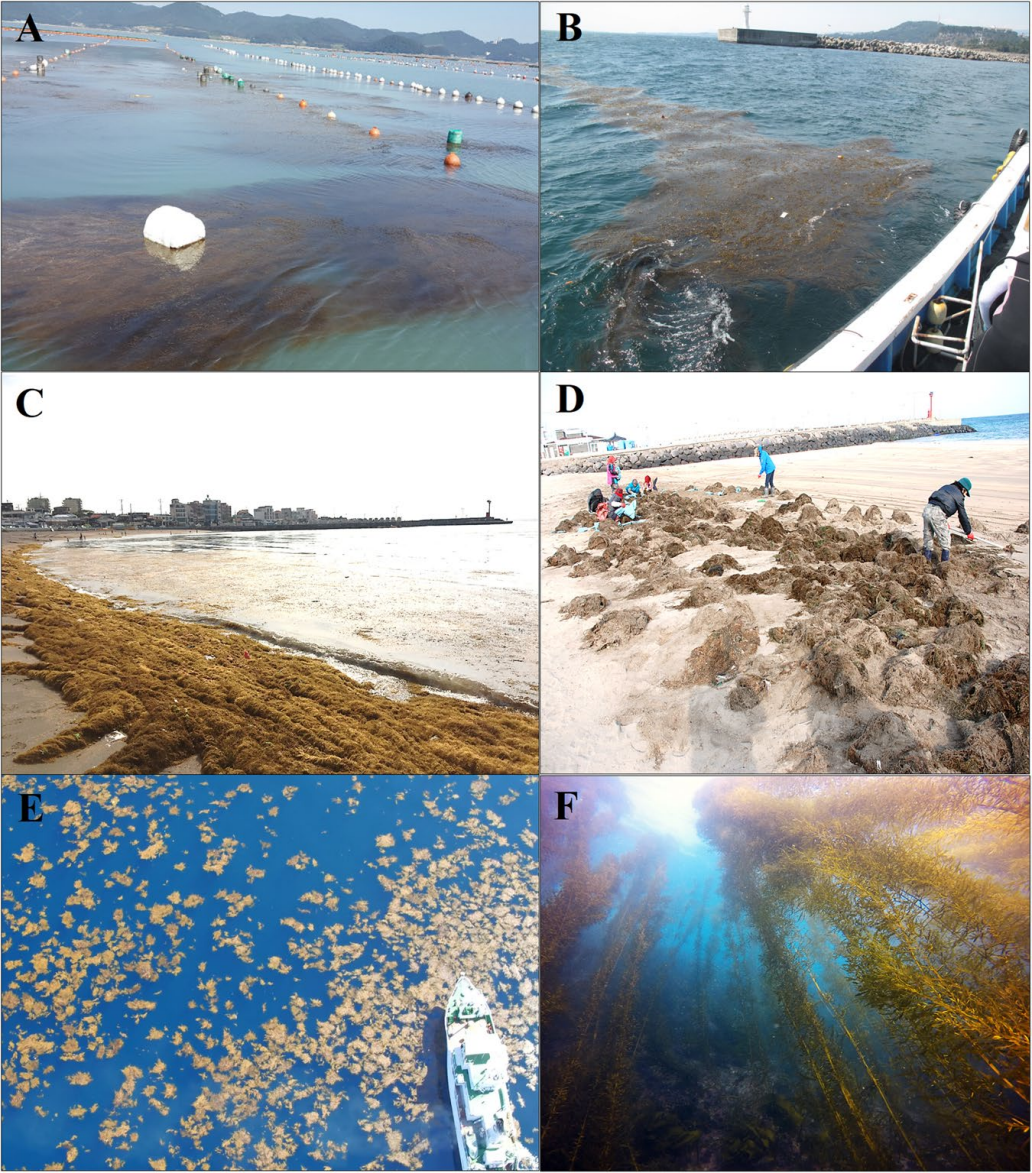
Golden tide seaweeds *Sargassum horneri* in Korean waters and the East China Sea (ECS). (**A**) Floating biomass stranded on *Porphyra* cultivation rafts in Haenam (HN) from the South Sea. (**B**) Floating biomass in seawater from Seogwipo (SGP) in Jeju Island. (**C**,**D**) *Sargassum horneri* stranded on shorelines in Jeju Island. (**E**) Floating biomass in seawater from the East China Sea (ECS). (**F**) Benthic population from the Munseom (MS) in Jeju Island.

*Sargassum horneri* is known to be the major golden-tide seaweed in the northwestern Pacific coasts including China, Japan and Korea^5,12^ as well as the northeastern Pacific coasts^7,13^. More recently, *S*. *horneri* that bloomed in the eastern coast of China has frequently drifted in a large biomass with oceanic currents and accumulated along the southern coast of Korea including Jeju Island, off the southernmost region of the mainland, and also the South and West Sea since 2015^5^ (Fig. 1A–E). The national wide concerns on the adverse impacts of the floating S. *horneri* populations on the coastal ecosystems in Korea have been growing particularly recently. Yet, the original sources of this drifting biomass in Korean waters remain unclear.

Despite its detrimental effects as the golden tide seaweed, S. *horneri* and also other *Sargassum* spp. in a natural state (e.g. when they grow as attached to hard substrata) form kelp forests (Fig. 1F) and play an important role in ecosystem functioning such as serving as spawning and nursery grounds for various marine species in coastal ecosystems^14^. *Sargassum horneri* is a valuable component in the coastal ecosystems, particularly for maintaining local biodiversity. This species is a predominant species among the genus *Sargassum* in the northwestern Pacific, which belongs to the order Fucales (fucoids). This species is dioecious with ellipsoidal pneumatocysts and has an annual life cycle with sexual reproduction using reproductive receptacles^15^. However, the reproductive mode of *S*. *horneri* in a floating state has never been examined, although an earlier study of floating *Sargassum* species in the Sargasso Sea suggested vegetative reproduction for their entire lives^16^.

Given the direction of oceanic currents (e.g. the Kuroshio Current) according to satellite image data, floating *S*. *horneri* populations in Korean waters are presumed to originate from certain areas in the east coast of China^5,9^. The Korean coast has a geological position that is primarily influenced by the Kuroshio Current as well as the Yellow Sea Warm Current and the Tsushima Warm Current that merge into the Kuroshio Current^17^. Because oceanic current systems are intricate and dynamic, it is, however, difficult to uncover the precise geographic origins of floating populations solely based on satellite images with the ocean currents.

Genetic analysis may provide a useful tool for identifying strains, and tracking geographic origins of nonindigenous populations^18^. Several phylogeographic and population genetic studies have been performed to identify species/strains and source populations and pathways of colonization or to understand the genetic diversity and population structure dynamics of several nonindigenous seaweed species, such as *Asparagopsis* spp.^19^ and *Sargassum natans*^20^ and also our study species, S. *horneri*^21^. A recent genetic survey of floating and benthic populations of *S*. *horneri* primarily focused on the east coast of China (the Yellow Sea) and sample sizes of Korean samples used for their study are limited (*N* = 1 for each of the five samples)^21^. A more recent study investigated the genetic composition of only floating samples of *S*. *horneri* exclusively from China’s east coast^22^. Hence, the phylogeographic/population structure and genetic diversity of drifting populations of *S*. *horneri* in Korean waters remain largely unknown. Also, the genetic structure of benthic populations of *S*. *horneri* on the Korean coast has not yet been studied.

In this study, by performing a phylogeographic analysis of mitochondrial DNA (mtDNA) cytochrome c oxidase III (*cox3*), we aimed to trace the geographic or genetic origins of 14 floating *S*. *horneri* populations to the Korean coasts including Jeju Island, the South Sea and the West Sea, and also in the East China Sea (ECS). We also analysed and compared the genetic structure between floating (*N* = 14) and benthic populations (*N* = 5) in Korean waters, based on seven nuclear microsatellite markers. Using floating populations collected at different time periods during a 2015–2018 year, we further tested whether there were changes in their genetic compositions over time. The results of our study will provide important insights into the golden-tide seaweeds in Korean waters and inform management efforts including the development of “*S*. *horneri* blooming forecasting system” that is now underway by the Korean government, which will help to prevent or mitigate ecological and economic damages on the Korean coastal ecosystems in the future.

## Methods

### Sample collection

**Figure 2:**
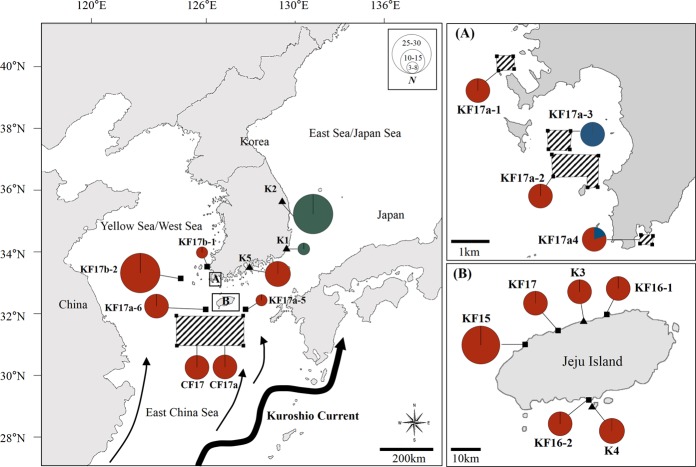
Map of sampling sites for floating and benthic populations of *Sargassum horneri* from the South Sea, the West Sea, the East Sea and Jeju Island along the Korean coast and also from the East China Sea (ECS), approximately 250 km off the Korean Peninsula. Floating populations (shown by small black-filled squares) were sampled from 13 different localities (the ECS population was sampled twice at different time periods in 2017) and benthic populations (small black-filled triangles) were sampled from five localities. The sampling sites of the ECS (CF17 and CF17a) and Haenam (HN; KF17a-1–4) are shown as marked areas with oblique lines connecting the points. (**A**) Haenam (HN; KF17a-1–4) from the South Sea. (**B**) Jeju Island, off the southernmost region of the mainland. This map also shows the geographical distributions of mtDNA *cox3* haplotypes of *S*. *horneri* that we determined. The area of the circle is proportional to individual numbers found for the respective haplotype and different colours denote different haplotypes (red: Hap 1, green: Hap 2, blue: Hap 3). Black arrow lines represent directions of the Kuroshio Current around Korean waters. Detailed information of each sampling locality is given in Table 1. Population abbreviations as in Table 1.

We sampled *S*. *horneri* individuals (*N* = 416) from 18 different localities along the Korean coasts including the South Sea, the West Sea and the East Sea as well as Jeju Island, which is off the southernmost region of the mainland, and also in the ECS from August 2015 to January 2018 (Table 1, Fig. 2). Sampling sites for floating populations (*N* = 14) included KF15, KF16-1, KF16-2, KF17, CF17, CF17a, KF17a-1, KF17a-2, KF17a-3, KF17a-4, KF17a-5, KF17a-6, KF17b-1 and KF17b-2. Of those, CF17 and CF17a were comprised of drifting populations from the ECS, approximately 250 km off the Korean Peninsula, and sampled twice from the same areas at different time periods in 2017 to test for the temporal genetic structure (Table 1, Figs 1E and 2). Those for benthic populations (*N* = 5) included K1 and K2 from the East Sea, K3 and K4 from Jeju Island, and K5 from the South Sea (Table 1, Fig. 2). Names of these sampling localities and sampling dates are given in Table 1. While whole *S*. *horneri* individuals attached to hard substrata were sampled by scuba-diving for ‘benthic’ samples, ‘floating’ samples were collected from drifting in seawaters, stranded on *Porphyra* cultivation rafts or stranded on shorelines (Table 1, Fig. 1).

**Table 1:** Information on sampling localities and periods, population codes and number of *Sargassum horneri* samples analysed in this study.

Sampling locality	Population code	Population type	Sample state	Latitude	Longitude	Sampling period	*N*_COX_	*N*_MSAT_
HJ (Hyeopjae)	KF15	Floating	Stranded on shorelines	33°23′38.88″N	126°14′23.02″E	Feb 2015	30	30
JJP (Jeju Port)	KF16-1	Floating	Stranded on shorelines	33°31′15.08″N	126°32′11.98″E	Mar 2016	10	10
SGP (Seogwipo)	KF16-2	Floating	Floating in seawater	33°13′48.40″N	126°34′1.09″E		15	30
SS (Sasu Port)	KF17	Floating	Stranded on shorelines	33°30′35.25″N	126°28′47.04″E	Feb 2017	10	30
ECS1 (East China Sea 1)	CF17	Floating	Floating in seawater	32°29′95″N 32°29″89″N 32°00′01″N 31°59′99″N 31°30′04″N 31°29′93″N	124°12′98″E 127°120′91′E 124°13′05″E 126°30′08″E 124°13′00″E 127°05′94″E		10	30
ECS2 (East China Sea 2)	CF17a	Floating	Floating in seawater			Apr–May 2017	10	30
HN (Haenam)-1	KF17a-1	Floating	Stranded on *Porphyra* cultivation rafts	34°21′52.6″N 34°21′52.6″N 34°22′03.5″N 34°22′03.5″N	126°27′42.4″E 126°28′10.3″E 126°28′10.3″E 126°27′42.4″E		7	7
HN-2	KF17a-2	Floating	Stranded on *Porphyra* cultivation rafts	34°19′13.2″N 34°19′40.9″N 34°19′40.9″N 34°19′25.9″N 34°19′25.9″N 34°19′13.4″N	126°30′39.6″E 126°30′39.9″E 126°29′48.0″E 126°29′48.0″E 126°30′25.4″E 126°30′25.4″E		11	13
HN-3	KF17a-3	Floating	Stranded on *Porphyra* cultivation rafts	34°20′16.3″N 34°20′16.3″N 34°20′02.3″N 34°20′02.3″N	126°29′29.9″E 126°30′11.5″E 126°30′11.5″E 126°29′29.9″E		10	24
HN-4	KF17a-4	Floating	Stranded on *Porphyra* cultivation rafts	34°18′00.9″N 34°18′00.9″N 34°17′52.2″N 34°17′52.2″N	126°32′03.1″E 126°32′07.6″E 126°32′07.6″E 126°32′03.1″E		10	28
SSL (South Sea; Line)	KF17a-5	Floating	Floating in seawater	33°00′00.0″N	127°24′00.0″E		6	6
JJSW (Jeju Southwest)	KF17a-6	Floating	Floating in seawater	33°01′27.7″N	125°44′49.0″E		8	30
SA (Sinan)	KF17b-1	Floating	Stranded on *Porphyra* cultivation rafts	34°48′21.7″N	125°59′06.3″E	Dec 2017–Jan 2018	5	5
WS (West Sea; Line)	KF17b-2	Floating	Floating in seawater	34°43′00.1″N 34°05′30.1″N 34°05′30.1″N	124°35′48.1″E 125°00′00.0″E 124°48′00.0″E		25	26
DBP (Daebyeon Port)	K1	Benthic	—	35°13′3″N	129°13′45″E	May 2016	3	3
DSP (Deoksan Port)	K2	Benthic	—	37°22′38″N	129°15′17″E	May 2016	26	25
YDA (Youngduam)	K3	Benthic	—	33°30′59.06″N	126°30′32.43″E	May 2016	5	30
MS (Munseom)	K4	Benthic	—	33°13′38″N	126°34′04″E	Mar 2016	15	29
YS (Yeosu)	K5	Benthic	—	34°45′00.07″N	127°39′20.46″E	Nov 2015	14	30
Total							230	416

“Floating” samples were collected from drifting in the seawaters, stranded on *Porphyra* cultivation rafts or stranded on shorelines, whereas “benthic” samples were obtained from intact *S*. *horneri* individuals attached to benthic substrata by scuba-diving (see Fig. 1). *N*_COX_: number of individuals sequenced for mtDNA *cox3*, *N*_MSAT_: average sample sizes across seven microsatellites genotyped.

Collected samples were rinsed using tap water and raked to remove epiphytic algae using a sterilized razor blade. All samples were then dried at 60 °C for 24 h and then ground using a TissueLyser II (QIAGEN, Mainz, Germany). Powdered samples were stored in a 1.5 ml microcentrifuge tube with silica gel and stored at −20 °C until genetic analysis.

### Mitochondrial (mt) DNA sequencing

Genomic DNA was extracted from pulverized leaf samples using i-genomic Plant DNA Extraction Mini Kit (iNtRON Biotechnology, Daejeon, Korea). A partial fragment (469 bp) of mtDNA *cox3* gene, previously shown to possess relatively higher polymorphism than chloroplast DNA markers^23^, was amplified for 230 samples using the published primers CAF4A and CAR4A^24^. Polymerase chain reaction (PCR) amplification was carried out in a reaction volume of 15 μl containing 1 × PCR buffer, 25 μM of each dNTP (Bio Basic Inc., Markham, ON, Canada), 0.6 μM of each of the forward and reverse primers, 0.2 units of Taq DNA polymerase (Thermo Fisher Scientific, Waltham, MA, USA) and approximately 5−10 ng of genomic DNA. RCR thermal cycling conditions comprised an initial denaturation at 94 °C for 3 min, followed by 35 cycles of denaturation at 94 °C for 20 s, annealing at 50 °C for 50 s and extension at 68 °C for 60 s, and a final extension at 68 °C for 10 min. PCR products were checked on 2% agarose gels stained with Redsafe (iNtRON). The amplified mtDNA were purified enzymatically with Exonuclease I (New England BioLabs, Ipswich, MA, USA) and Shrimp Alkaline Phosphatase (New England BioLabs). The purified mtDNA fragments were subjected to direct sequencing only in the reverse direction using the same reverse primers as in the PCR and the BigDye Terminator 3.1 Cycle Sequencing Ready Reaction Kit in an ABI 3730xl automated DNA sequencer (Applied Biosystems, Foster City, CA, USA). The DNA sequences were edited using CHROMAS Lite v2.1.1 computer software and aligned with Clustal W^25^ implemented in BioEdit v7.2.5^26^, and finally verified manually.

### Microsatellite genotyping

Seven polymorphic nuclear microsatellite loci were amplified with the published primers including SHORN18, SHORN26, SHORN27, SHORN30, SHORN31, SHORN34 and SHORN 41^27^. Each of the forward primers was labelled with a fluorescent dye (FAM, HEX and TET). PCR reactions were conducted as described for the mtDNA *cox3* gene. PCR cycling conditions were carried out as suggested in a previous study^27^. The PCR products were electrophoresed on an ABI 3730xl automated DNA sequencer (Applied Biosystems). Fragment sizes were determined with the ROX 500 bp size standard (ABI) using GENEMAPPER software v5.0 (Applied Biosystems).

### Statistical analyses

#### Phylogeographic analysis

To trace original genetic sources of floating populations to the Korean coast, and also to determine phylogeographic relationships between mtDNA haplotypes of *S*. *horneri*, partial sequences of mtDNA *cox3* (Accession Nos.: JF461002-JF461052; H1–H51) representing the northwestern Pacific lineages including Chinese and Japanese clades were retrieved from GenBank^17^. Only 45 previous haplotype sequences were used for the final analysis, as the 51 haplotypes were merged into 45 haplotypes due to adjustments to a shorter length of 469 bp. The best nucleotide substitution model was tested using initial searches according to the AICc criterion with jModelTest v2.1.7^28^, which selected the Jukes-Cantor (JC) model. The phylogenetic tree was then reconstructed with maximum likelihood (ML) method using as implemented in Mega v7.0^29^. Sequences of *S*. *muticum* as outgroup were retrieved from GenBank (Accession Nos.: AB430582.1) and statistical support was estimated by 1000 bootstrap replicates.

Phylogenetic analysis was also conducted with a neighbour joining (NJ) approach, based on microsatellite allelic variation. *D*_A_ distances among the 19 samples were calculated using POPTREE2^30^.

#### Population genetic analysis

To investigate differences in levels of microsatellite diversity among the 19 samples of *S*. *horneri*, the mean number of alleles per locus (*N*_A_), observed (*H*_O_) and expected (*H*_E_) heterozygosity, and allelic richness (AR) corrected for unbalanced sample sizes among the samples were calculated using GENEPOP v4.3^31^ and FSTAT v2.9.3.2^32^. The AR values were estimated after excluding the samples of KF17a-1, KF17a-5, KF17b-1 and K1 because of insufficient sample sizes (*N* < 10). A Mann-Whitney *U* test was performed to examine whether there was a significant difference in the level of AR between floating (*N* = 11) and benthic (*N* = 4) populations of *S*. *horneri*. The four samples with limited sample sizes were not included for downstream analyses, with the exception of AMOVA, STRUCTURE, FCA and phylogenetic analyses (see below). Multilocus tests for Hardy-Weinberg equilibrium (HWE) and linkage disequilibrium (LD) tests for genotypes among pairs of the seven loci were also performed in GENEPOP. The 95% significance levels for every exact test for both HWE and LD were corrected using a Bonferroni correction. We also tested for the presence of null alleles across the seven loci using MICROCHECKER v2.2.3^33^ with 1000 randomization at the 95% confidence level.

To assess the degree of genetic differentiation between and within floating and benthic populations of *S*. *horneri*, calculation of pair-wise *F*_ST_ estimates among the 15 populations as well as exact tests for population differentiation were performed using GENEPOP. The 95% significance levels for the pairwise comparisons were adjusted using a Bonferroni correction. Hierarchical analysis of molecular variance (AMOVA) was performed in ARLEQUIN v3.5.1^34^ to assess temporal genetic structure among floating samples from the different sampling periods, based on microsatellites. The temporal AMOVA was performed by classifying the 14 floating populations into five different groups according to sampling periods (Feb 2015, Mar 2016, Feb 2017, Apr–May 2017 and Dec 2017–Jan 2018).

The genetic structure among and within floating and benthic populations was further analysed using an individual-based Bayesian population assignment approach implemented in STRUCTURE v2.3.4^35^ under a model of admixed ancestry among populations and correlated allele frequencies with no *a priori* information on the geographic origins of the samples. STRUCTURE calculates a likelihood score when the data are forced into a given number of genetic clusters (*K*) = 1–19. We applied 10 iterations, with 10,000 burn-in steps followed by 100,000 Markov Chain Monte Carlo (MCMC) generations. STRUCTURE analyses were also carried out separately for floating populations and also for benthic populations at *K* = 1–14 and *K* = 1–5, respectively. The most likely number of genetic clusters (*K* value) was determined by using the Δ*K* method^35^ implemented in the web-based tool STRUCTURE HARVERSTER (http://taylor0.biology.ucla.edu/structureHarvester/)^36^, based on the rate of change in the probability of data between successive *K* values. In addition, factorial correspondence analysis (FCA) based on genetic relationships between individuals with multi-locus genotypes was conducted using GENETIX v4.05.2^37^.

## Results

### Phylogeographic analysis

We found only three mtDNA *cox3* haplotypes from a total of 230* S*. *horneri* individuals that we sequenced. While the 17 populations including 14 floating (KF15, KF16-1, KF16-2, KF17, CF17, CF17a, KF17a-1, KF17a-2, KF17a-3, KF17a-4, KF17a-5, KF17a-6, KF17b-1 and KF17b-2) and three benthic (K3, K4 and K5) populations shared an identical haplotype (Hap 1), the remaining two benthic populations (K1 and K2) from the East Sea shared another unique haplotype (Hap 2) (Fig. 2). Of the 14 floating populations, two populations, such as KF17a-3 (haplotype frequency: 1.0) and KF17a-4 (0.2) from the South Sea possessed a haplotype (Hap 3) that did not exist elsewhere (Fig. 2).

The Hap 1 was found to be identical to the previously determined haplotype (H7) that was observed in populations exclusively from Zhoushan, Zhejiang province across nearly entire east coasts of China and also from the western coast of Japan (Supplementary Fig. S1)^17^. The Hap 2 was also the same as a previously identified haplotype (H39) that was predominantly distributed in the northeastern coast of Japan (Supplementary Fig. S1)^17^. The Hap 3, which has never been detected before, was found only in the populations of KF17a-3 and KF17a-4 across all the populations analysed in this study (Fig. 2, Supplementary Fig. S1).

### Microsatellite diversity

**Figure 3:**
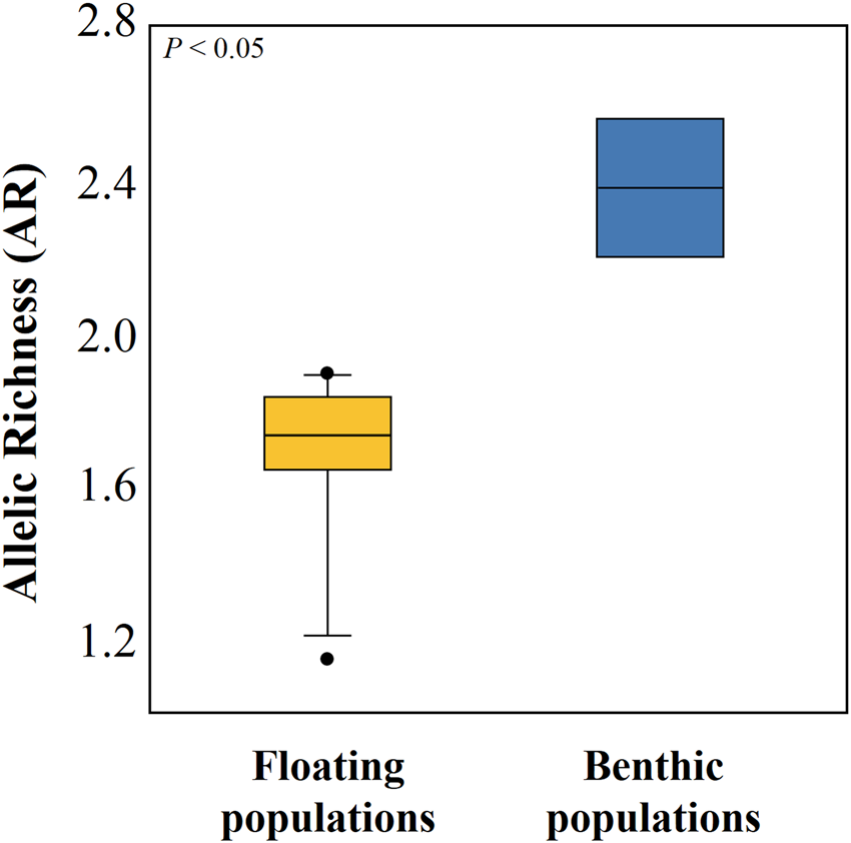
A box plot illustrating a difference in the level of genetic diversity (allelic richness; AR) between floating (*N* = 11) and benthic (*N* = 4) populations. The level of AR was significantly higher for Korean benthic populations than for floating populations (Mann-Whitney *U* = 0.000, *P* = 0.004). The horizontal line within the box is the median, the upper and lower boundaries of the box mark the 25^th^ and 75^th^ percentiles and the whiskers mark the minimum and maximum values.

The estimated frequencies of null alleles at the seven loci ranged from −0.11 (SHORN41) to 0.09 (SHORN34), indicating a low probability of null alleles. Microsatellite diversity indices *N*_A_, *H*_E_, *H*_O_, AR and HWE *P*-values within the 19* S*. *horneri* populations were estimated and are summarized in Table 2. Four Korean benthic populations (after excluding the K1 population) showed a significantly higher level of AR than 11 floating populations (excluding the KF17a-1, KF17a-5 and KF17b-1) (Mann-Whitney *U* = 0.000, *P = *0.004; Fig. 3). Tests of genotypic LD for the entire pooled population showed no significant association of alleles between the seven loci except for between SHORN26 and SHORN31, SHORN26 and SHORN30, and SHORN26 and SHORN34 (*P* < 0.05).

**Table 2:** Summary of genetic diversity statistics in 19 populations of *Sargassum horneri* in Korean waters as well as the East China Sea (ECS) at seven microsatellite markers. *N*: sample size, *N*_A_: mean number of alleles across loci, *H*_E_: expected heterozygosity, *H*_O_: observed heterozygosity, AR: allelic richness, HWE: *P* values for multilocus tests for Hardy-Weinberg Equilibrium.

Population	*N*	*N*_A_	*H*_E_	*H*_O_	AR	HWE
KF15	30	2.00	0.39	0.55	1.89	*
KF16-1	10	1.86	0.37	0.63	1.83	*
KF16-2	30	1.71	0.33	0.61	1.71	*
KF17	30	2.00	0.23	0.39	1.64	*
CF17	30	2.00	0.33	0.52	1.87	*
CF17a	30	1.86	0.29	0.42	1.82	*
KF17a-1	7	1.14	0.08	0.14	—	—
KF17a-2	13	1.14	0.07	0.14	1.14	*
KF17a-3	24	1.86	0.19	0.27	1.65	*
KF17a-4	28	2.00	0.17	0.17	1.74	*
KF17a-5	6	1.43	0.23	0.43	—	—
KF17a-6	30	1.57	0.22	0.43	1.46	*
KF17b-1	5	1.43	0.24	0.43	—	—
KF17b-2	26	1.86	0.26	0.43	1.73	*
K1	3	1.43	0.17	0.19	—	—
K2	25	3.00	0.28	0.33	2.19	NS
K3	30	2.86	0.27	0.28	2.20	*
K4	29	3.43	0.35	0.36	2.54	*
K5	30	3.29	0.44	0.42	2.56	*

Four populations (KF17a-1, KF17a-5, KF17b-1 and K1) with insufficient sample sizes (*N* < 10) were excluded from some of the genetic diversity statistics (e.g. AR, HWE). Population abbreviations as in Table 1. *P < 0.05 after a Bonferroni correction applied; NS: not significant.

### Genetic differentiation and population structure

Pairwise *F*_ST_ estimates for the microsatellites between the 15 populations revealed significant genetic differentiation except eight comparisons, which were always from those within floating populations (Table 3). However, weak but some statistically significant genetic differentiation was observed within floating populations, suggesting that they may have originated from different genetic sources.

**Table 3:** Pairwise genetic differentiation (*F*_ST_) between 15 populations of *Sargassum horneri* in Korean waters and the East China Sea (ECS), based on seven microsatellite markers.

	KF15	KF16-1	KF16-2	KF17	CF17	CF17a	KF17a-2	KF17a-3	KF17a-4	KF17a-6	KF17b-2	K2	K3	K4
KF16-1	0.02													
KF16-2	0.03	0.00												
KF17	**0**.08*	**0**.14*	**0**.11*											
CF17	**0**.06*	**0**.10*	**0**.11*	**0**.07*										
CF17a	**0**.08*	**0**.14*	**0**.13*	**0**.03*	**0**.05*									
KF17a-2	**0**.24*	**0**.36*	**0**.29*	**0**.29*	**0**.23*	**0**.20*								
KF17a-3	**0**.40*	**0**.48*	**0**.44*	**0**.42*	**0**.40*	**0**.38*	**0**.64*							
KF17a-4	**0**.21*	**0**.28*	**0**.24*	**0**.20*	**0**.18*	**0**.13*	0.03	**0**.51*						
KF17a-6	**0**.08*	**0**.15*	**0**.12*	0.00	**0**.07*	**0**.03*	**0**.29*	**0**.46*	**0**.20*					
KF17b-2	**0**.07*	**0**.13*	**0**.11*	0.01	**0**.05*	0.00	**0**.24*	**0**.42*	**0**.16*	0.01				
K2	**0**.31*	**0**.33*	**0**.33*	**0**.35*	**0**.33*	**0**.34*	**0**.53*	**0**.49*	**0**.47*	**0**.36*	**0**.34*			
K3	**0**.29*	**0**.33*	**0**.31*	**0**.28*	**0**.27*	**0**.23*	**0**.47*	**0**.31*	**0**.39*	**0**.30*	**0**.26*	**0**.40*		
K4	**0**.35*	**0**.34*	**0**.36*	**0**.37*	**0**.36*	**0**.34*	**0**.49*	**0**.26*	**0**.43*	**0**.39*	**0**.36*	**0**.37*	**0**.25*	
K5	**0**.29*	**0**.29*	**0**.33*	**0**.31*	**0**.27*	**0**.26*	**0**.39*	**0**.16*	**0**.33*	**0**.33*	**0**.29*	**0**.33*	**0**.23*	**0**.20*

Significant pairwise and *P* values are shown in bold (*P < 0.05) after the Bonferroni correction. Population abbreviations as in Table 1.

The temporal AMOVA based on microsatellites revealed absence of genetic structure among the five temporal groups (*F*_CT_ = 0.10, *P* = 0.19), but a highly significant genetic structure within temporal groups (*F*_SC_ = 0.23, *P* < 0.001) (Table 4). These findings indicate that more genetic variation exists within floating populations from the same sampling periods than from different periods. The overall genetic variation among samples whatever the groups was also significant (*F*_ST_ = 0.30, *P* < 0.001).

**Table 4:** Result of AMOVA (Analysis of Molecular Variance) for the 14 floating populations of *Sargassum horneri* based on seven microsatellite markers.

	*df*	% variation	Fixation indices	*P*-value
Among temporal groups	4	9.64	*F*_CT_ = 0.10	0.19
Among populations within temporal groups	8	20.80	*F*_SC_ = 0.23	<0.001
Within populations	425	69.55	*F*_ST_ = 0.30	<0.001

The temporal AMOVA was performed by classifying the floating populations into five different groups according to sampling periods (Feb 2015, Mar 2016, Feb 2017, Apr–May 2017 and Dec 2017–Jan 2018; see “Methods” section in more details).

**Figure 4:**
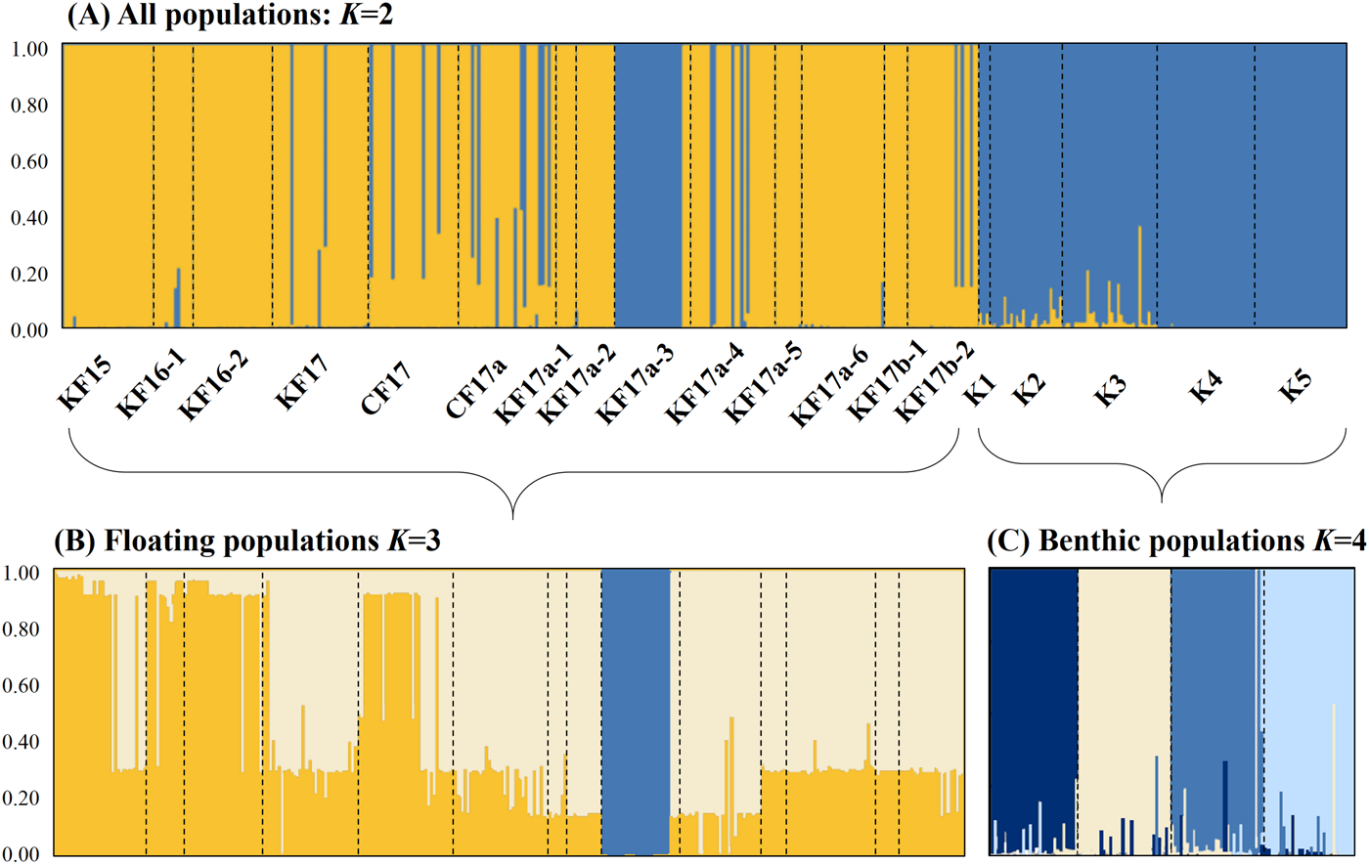
Results of genetic structure using a Bayesian population assignment test with STRUCTURE for the study populations of *Sargassum horneri*, based on seven microsatellite markers. Each individual is represented along the X-axis, and the Y-axis denotes the probability of that individual belonging to each of the genetic clusters. (**A**) Analyses of population structure for the all 19 populations combined. The most likely number of genetic clusters after Delta K Evanno’s correction corresponds to *K* = 2. Yellow colour represents floating populations, and blue colour represents benthic populations including the floating population of KF17a-3. (**B**) Analyses of population structure only for the 14 floating populations. The most likely number of genetic clusters after Delta K Evanno’s correction corresponds to *K* = 3 but the alpha (α) value was not stabilized (when the KF17a-3 population excluded), because of the very similar genetic structure among the 13 floating populations. (**C**) Analyses of population structure for the five benthic populations. The most likely number of genetic clusters after Delta K Evanno’s correction corresponds to *K* = 4.

**Figure 5:**
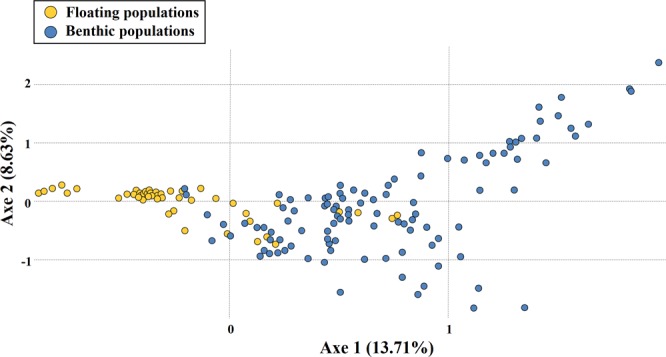
Results of factorial correspondence analysis (FCA) showing multivariate relationships among the 19 populations of *Sargassum horneri* based on microsatellite allelic variation. Yellow circles represent 14 floating populations, and those in blue colour represent Korean benthic populations. Floating samples showing more close relationships with benthic genotypes (around the middle in the graph) are from the KF17a-3 population.

STURCTURE analyses based on the microsatellites revealed the most likely two genetic clusters (*K* = 2) that were comprised of 13 floating (except KF17a-3) and five benthic populations for each separate cluster (Fig. 4A), which was further confirmed by FCA (Fig. 5). STRUCTURE showed that the floating KF17a-3 population was clustered with benthic populations except for three individuals being clustered with floating populations, suggesting that this particular population harboured individuals with both benthic and floating genotypes. When STRUCTURE analysis was performed only for the 14 floating populations, the most likely number of genetic clusters corresponded to three genetic clusters, but the 13 populations except KF17a-3 showed homogeneous distributions of individual genotypes, in which all individuals were assigned to approximately equal proportions of the inferred two genetic clusters, implying they are genetically virtually indistinguishable (Fig. 4B). When STRUCTURE analysis was performed only for the five Korean benthic populations, four genetic clusters were best fitted the data with two populations (K1 and K2) from the East Sea grouped together and the other three populations their own groups (Fig. 4C).

**Figure 6:**
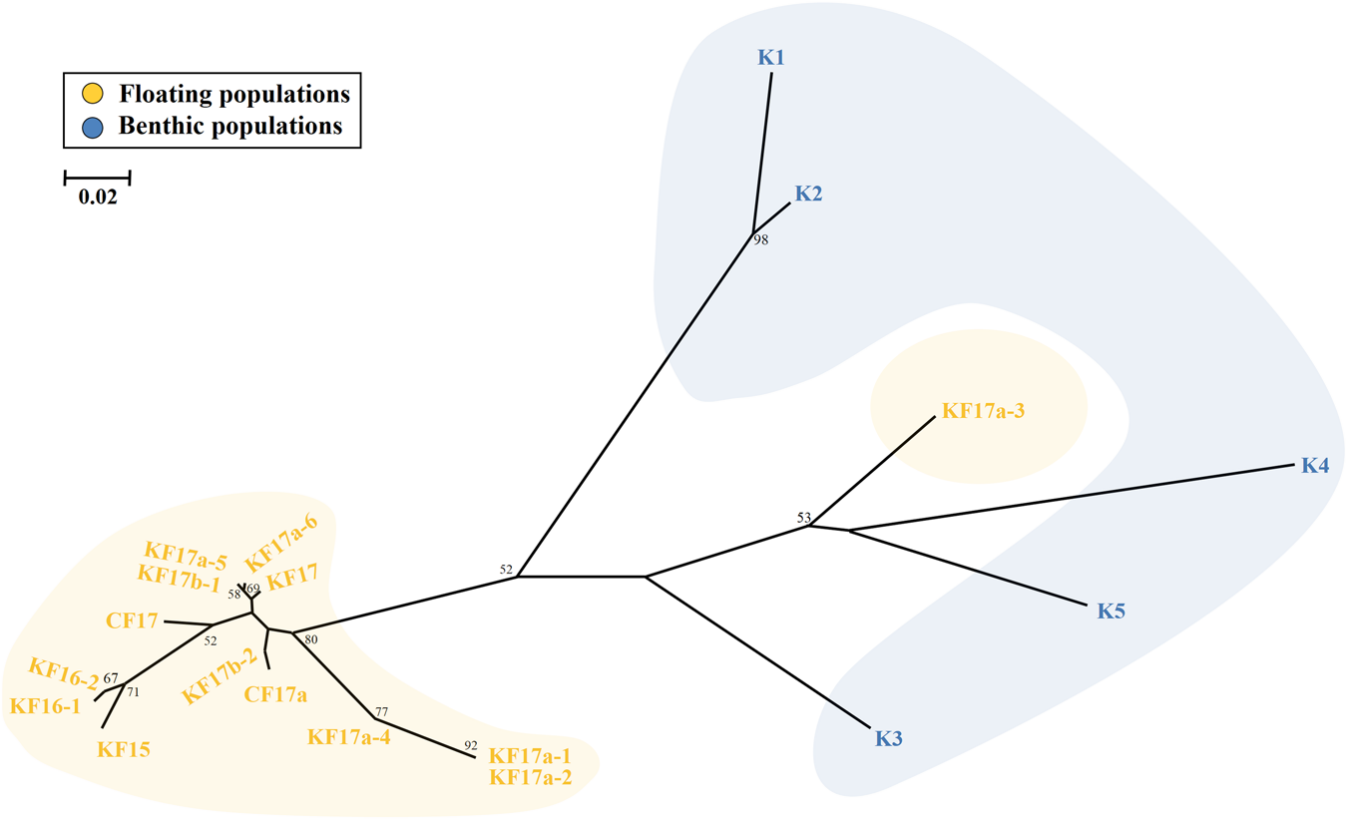
The neighbour-joining (NJ) tree based on microsatellites using *D*_A_ distances among the 19 populations of *Sargassum horneri*. The floating and benthic populations form different phylogenetic clusters except for the KF17a-3. Among the five benthic populations, two populations (K1 and K2) from the East Sea formed a distinct cluster. Overall, the benthic populations represent relatively greater genetic distances among the samples than the floating populations. Only bootstrap support values >50% are shown and the scale bar denotes genetic distance. Population abbreviations as in Table 1.

Our phylogenetic analysis of microsatellites based on *D*_A_ distances among the 19 samples showed that the floating and benthic populations of *S*. *horneri* formed two separate clades as for the results of STRUCTURE and FCA (Fig. 6). Again, the KF17a-3 population formed a cluster with benthic clades. For the five benthic populations, two populations (K1 and K2) from the East Sea formed one clade and the remaining three populations another lineage. The 13 floating populations (except KF17a-3) represented relatively closer genetic distances among the samples compared to Korean benthic populations.

## Discussion

### The origin(s) of the floating *S*. *horneri* populations in Korean waters

Information on geographic or genetic origins (sources) of the floating *S*. *horneri* biomass provides an important insight into advancing our understanding of the recurrent golden tides^21,22^. Often a drifting seaweed biomass is hardly predictable to gauge their movement direction, as they are not usually distributed in an equal amount for every year and also ocean currents sometimes dramatically change their dispersal direction, owing to environmental factors such as wind direction^38^. A central goal of our study was to track geographic or genetic origins of floating *S*. *horneri* populations on the southern coast of Korea, based on phylogeographic analysis of mtDNA marker by utilizing existing sequence dataset available. The Hap 1 was found to be predominantly distributed along the western coast of Japan and also exclusively in the Zhoushan region across almost entire east coasts of China in a previous study^17^. Moreover, a unique haplotype to the East Sea, Hap 2, is turned out to be the same as the haplotype detected from the northeastern coast of Japan (e.g. Aomori, Iwate and Miyagi prefectures)^17^. The Hap 3 is only found in the floating samples of KF17a-3 and KF17a-4 from the South Sea across all the populations examined, which has never been detected in previous studies.

Our findings that although most Korean benthic samples also possess the same haplotype, the floating *S*. *horneri* individuals share the Hap 1, which was found only in the Zhoushan, Zhejiang province across the east coasts of China (H7 in their study)^17^ might suggest that the populations from this area represent geographic or genetic sources of floating populations in Korean waters. This hypothesis is plausible, especially given influential ocean current systems (e.g. the Kuroshio Current) around this region^3,39^. Although the Hap 1 is also relatively common along the western coast of Japan, populations from this area are highly unlikely to serve as source populations to the Korean coast, considering the direction of major oceanic currents, such as the Kuroshio Current and the Tsushima Warm Current nearby. Therefore, given the shared distributions of mtDNA *cox3* haplotypes between the Zhoushan, Zhejiang province and floating populations in Korea as well as the oceanic circulation systems in this region, the drifting *S*. *horneri* biomass in Korean waters may have been originated from this particular region on the southeastern coast of China. A previous study demonstrated that drifting *S*. *horneri* rafts of origins of Zhejiang province can disperse up to the eastern ECS under the effect of the Kuroshio Current^12^. A more recent study provides genetic evidence supporting the hypothesis that floating *S*. *horneri* rafts in the Yellow Sea might be of origins of Zhejiang region^40^. Given those earlier work and satellite data, our findings may not be unexpected, but the results of our study can provide direct (genetic) evidence supporting the hypothesis that floating golden tide seaweeds from the southeastern coast of China actually travel up to the Korean coast with ocean currents^5^.

Although mtDNA *cox3* gene has frequently been used as a molecular marker to examine intraspecific polymorphism for *S*. *horneri*^17,22^, variation in the *cox3* is not sufficiently enough to discriminate between benthic and floating populations in our study. Hence, further studies using more polymorphic markers with additional samples from “benthic” populations on the east coast of China, particularly from the Zhoushan region will be required to determine the origins of floating *S*. *horneri* biomass more precisely. Currently, we are developing molecular markers that can differentiate between floating and Korean benthic populations by exploring organelle genomes, but it may not be feasible because mt- and cp-DNA genomes of *Sargassum* species are known to be relatively conservative^23^. We are also applying recently developed microsatellite markers, which are assumed to be more polymorphic than ones used in this study, to testing whether they will provide a better resolution for elucidating the genetic structure of floating samples^41^. However, more extensive sampling, covering particularly from China’s east coast is the most critical for determining the origins, movement patterns and population structure of floating *S*. *horneri* biomass. For this, collaborative efforts, such as sharing *S*. *horneri* (DNA) samples for genetic analysis, with a counterpart of China through establishing a research consortium will help to advance our current knowledge of the golden tides in this region.

### Differences in genetic structure between benthic and floating populations

Since the *cox3* gene did not house enough variation to discriminate between benthic and floating populations, we applied more polymorphic microsatellites as an additional marker-set to assessing the genetic structure between these samples. Although a mtDNA molecule has been useful as a phylogeographic marker for inferring population or evolutionary histories of seaweed species, hyper-variable microsatellite regions may be more appropriate for understanding ‘contemporary’ processes of seaweed populations as they usually possess higher mutation rates^17^. Differences in the levels of polymorphism between mtDNA and microsatellite markers can also be linked to the pattern of maternal inheritance of mitochondria^42^. Mitochondrial inheritance is suggested to be maternal in brown algae^43^, although this has not been confirmed for *S*. *horneri*. The results of microsatellites show that floating *S*. *horneri* populations are clearly genetically different from benthic populations (except the KF17a-3 population), as suggested by our multifaceted-lines of analyses such as pairwise *F*-statistics, STRUCTURE, FCA and also NJ phylogeny. When we investigated all populations combined, strong genetic structure was found between floating and benthic samples, as indicated by two genetic clusters determined by STRUCTURE and FCA analyses. Unexpectedly, the floating KF17a-3 population, which was collected from stranded biomass on *Porphyra* cultivation rafts, shares a gene pool with Korean benthic populations, except for the three individuals that belong genetically to floating populations (Fig. 4A). We hypothesize that some Korean benthic individuals surrounding this area were naturally detached from the benthic substrata and detached thalli floated on the sea surface and stranded *Porphyra* farm rafts^12^. This population is indeed comprised of a mixture of 21 genetically ‘benthic’ and three genetically ‘floating’ individuals. According to results of our STRUCTURE analysis, none of the KF17a-3 samples shows a sign of genetic admixture between floating and benthic genotypes. The hypothesis of the natural detachment of *S*. *horneri* individuals from local benthic populations is plausible, given that this species is distributed widely around this region of the Haenam (HN)^44^. Also, naturally detached *S*. *horneri* thalli due to wave action were sometimes observed in this area (S. Kim, personal observation). Nevertheless, a further study with benthic samples from those particular sites will be needed to substantiate our hypothesis.

When benthic and floating populations analysed separately, benthic populations show considerable genetic structure with four genetically unique groups, whereas floating populations had similar genetic structure with a rather, single genetically homogeneous group except for the KF17a-3 population. The Korean benthic populations are genetically more diverse and substantially divergent to one another with their own genetic integrities: one represents the East Sea (K1 and K2), one the south of Jeju Island (K3), one the north of Jeju Island (K4) and one the South Sea (K5). The observed spatial variation in the genetic structure among the benthic populations of *S*. *horneri* most likely results from limited contemporary gene flow occurring across these geographically disconnected populations^42^.

On the other hand, the drifting *S*. *horneri* rafts represent indistinguishable genetic structure, at least in part due to the overall low levels of genetic diversity within floating samples, possibly caused by the vegetative growth during their floating life stage after detached from the benthic substrata^16^. Although drifting *Sargassum* species in the Sargasso Sea was shown to be solely vegetatively reproduced^16^, whether our study species, *S*. *horneri* uses the same reproductive strategy in a floating state is unknown. Therefore, the reproductive mode of floating *S*. *horneri* will need to be evaluated to test this hypothesis. Since 2011, the Chinese government has run a large-scale transplantation and farming project for the purpose of the construction of *S*. *horneri* forests around Zhoushan, Zhejiang province, which span an area of approximately 860,000 square kilometres^45^. Most seaweed farming exercises with a limited number of parental strains, which ensues the loss of genetic diversity in the entire Zhoushan population of *S*. *horneri*.

While floating populations have continuously been introduced into Korean waters, particularly recently, the observed lack of gene flow between these and Korean benthic populations can be explained by several hypotheses. First, based on previous studies that found differences in the timing of reproduction in *S*. *horneri* populations due to the temperature gradient of seawater along the latitude^15,46^, gene flow between floating and benthic populations might be hampered because of the differences in the timing of maturation (e.g. receptacle formation), given approximately one-to-three degree differences in the latitude between Zhoushan region of China and southern coasts of Korea. Another possible explanation would be that the establishment of southern floating populations on the Korean coast may be hindered because already established Korean benthic populations preoccupy space and resources, having negative impacts on their successful establishment (i.e. priority effect)^47^. Lastly, we could not simply sample admixed individuals between floating and benthic populations, although they are present. A more detailed analysis of benthic populations from areas where drifting assemblages are frequently observed will allow to validate the presence or absence of ‘introduced’ or ‘admixed’ individuals.

Based on a previous study^21^, we predicted that floating populations from the same periods will be genetically more uniform than from different periods, since they are more likely to be originated from the same origins. However, weak but some significant genetic variation was observed within floating populations particularly from the same periods, suggesting there may be multiple genetic sources being present. These findings imply that drifting populations in Korean waters at different locations from the same periods would consist of genetically dissimilar individual clusters, which may have been originated from populations with genetically different multiple patches within geographic origin(s). Therefore, the most plausible scenario would be that after *S*. *horneri* individuals were detached from genetically distinct natural beds in the southeastern coast of China (e.g. Zhoushan, Zhejiang province), albeit low genetic diversity apparent, they may undergo a rapid vegetative growth in the ocean during their drifting lives^16^ and genetically heterogeneous massive seaweeds may then subsequently drift into the Korean coast^48,49^. Our temporal AMOVA seems to support this hypothesis that the genetic variation observed among the five temporal groups was considerably lower than within the groups. A recent study of mtDNA marker suggests that the floating golden tide populations in the Yellow Sea comprise at least two genetic lineages or sources^22^, supporting the hypothesis that multiple genetic sources exist for floating *S*. *horneri* rafts in Korean waters in this study.

Yet, some genetic variation exists within temporally varying floating samples. For example, the KF17 floating population in Jeju Island in 2017 differs in the genetic structure from previous floating populations during a 2015–2016 year (*F*_ST = _0.08−0.14, *P* < 0.05; Table 3). Also for the ECS drifting population off the ocean, the gene pool appears to be variable over even a short period of time, given significant differentiation detected between temporal samples that were only two months apart (Feb and Apr in 2017; CF17 vs. CF17a: *F*_ST = _0.05, *P* < 0.05), suggesting there may be of different genetic sources. Alternatively, assuming that drifting *S*. *horneri* individuals could undergo sexual reproduction, the considerable temporal variation can be interpreted as their low effective population sizes (*N*_e_) that are small enough to be subject to genetic drift (‘sweepstakes’ hypothesis^50^). In other words, the reproductive success of the ECS population of *S*. *horneri* may be like a sweepstakes, in which only a few individuals contribute to producing offspring recruit to the whole population, while a majority do not yield offspring at all^51^.

In conclusion, although precise genetic sources of the floating *S*. *horneri* populations in Korean coastal waters could not be firmly identified, our analyses suggest that the floating populations might originate from Zhoushan, Zhejiang province on the southeastern coast of China. Although previous studies already suggested that drifting *S*. *horneri* biomass around Zhejiang province can disperse up to the eastern ECS under the Kuroshio Current^12^, our study provides direct evidence confirming this hypothesis. Moreover, we find that substantial differences exist in genetic structure between floating and benthic populations. While the Korean benthic *S*. *horneri* populations are strongly genetically divergent to one another, the floating ones are generally genetically homogeneous, albeit some differentiation detected especially from the same periods. The results suggest that the floating populations might have multiple genetic sources within origin(s).

For management efforts, we suggest some possible controls to avoid or minimize damages on the Korean coast, based on our findings. First, drifting *S*. *horneri* rafts can be removed directly from the ECS prior to their arrival to the Korea coast, if the source areas and the timing of golden tide blooming are ascertained. This idea may not be unrealistic, considering a government-funded project of ‘the development of *S*. *horneri* removal system off the ocean’ launched in 2018 in Korea. Second, establishing an international consortium with counterparts of China and possibly Japan for making cooperative efforts, such as sharing information on the origins/timing as well as the magnitude of *S*. *horneri* blooming *in situ* will be effective to forecast a particular golden tide in Korean waters. Third, applying floating *S*. *horneri* biomass as potentially valuable industrial resources, such as food, medicated or agricultural fertilizers is also important for developing the effective management^2^. Lastly, once floating golden tide seaweeds introduced to the Korean coast, developing a molecular-based fast and accurate detection method is important for their rapid removal. For this, constructing a genetic database for Korean benthic populations will be needed. This study, to our knowledge, first provides genetic information on floating and benthic populations of *S*. *horneri* in Korea and will inform management efforts including “*S*. *horneri* blooming forecasting system”, which helps to protect and mitigate ecological damages on the Korean coastal ecosystems.

## Supplementary information

Supplementary Figure S1

## Data Availability

Mitochondrial DNA sequences can be accessed via GenBank with accession numbers MK695984-MK695986 (https://ncbi.nlm.nih.gov/nuccore). The microsatellite data for the population samples of *Sargassum horneri* are available within the published article.
