# Supplementary material for: The origin and population genetic structure of the ‘golden tide’ seaweeds, Sargassum horneri, in Korean waters
Source: Sci Rep. 2019 May 23;9:7757. doi: 10.1038/s41598-019-44170-x (PMC6533256; doi:10.1038/s41598-019-44170-x)

**The origin and population genetic structure of the ‘golden tide’ seaweeds, *Sargassum horneri*, in Korean waters**

**Seo Yeon Byeon^1^, Hyun-Ju Oh^2^, Sangil Kim^2^, Suk Hyun Yun^2^, Ji Hyoun Kang^3^, Sang Rul Park^4^*, Hyuk Je Lee^1^***

^1^ Molecular Ecology and Evolution Laboratory, Department of Biological Science, College of Science and Engineering, Sangji University, Wonju, 26339, Republic of Korea

^2^ Oceanic Climate and Ecology Research Division, National Institute of Fisheries Science, Busan, 46083, Republic of Korea

^3^ Korean Entomological Institute, Korea University, Seoul, 02841, Republic of Korea

^4^ Estuarine and Coastal Ecology Laboratory, Department of Marine Life Sciences, Jeju National University, Jeju, 63243, Republic of Korea

Authors for Correspondence

Hyuk Je Lee (H.J.L.)

E-mail: [hyukjelee@sangji.ac.kr](mailto:hyukjelee@sangji.ac.kr)

Sang Rul Park (S.R.P.)

E-mail: [srpark@jejunu.ac.kr](mailto:srpark@jejunu.ac.kr)

**Supplementary Figure S1.** Phylogenetic relationships among 48 mtDNA *cox3* haplotypes [previously determined 45 haplotypes^17^ plus three haplotypes (Hap 1−3) that were identified in this study] of *S. horneri* in the northwestern Pacific including Korea, China and Japan. Numbers at the branches represent the bootstrap support for maximum likelihood (ML) and only those >50% are shown. Sequences of previously detected *cox3* haplotypes representing Chinese and Japanese lineages were retrieved from GenBank (Accession Nos.: JF461002-JF461052; H1−H51). The 51 detected haplotypes were merged into 45 haplotypes, as they were adjusted to a shorter length of 469 bp [excluding JF461040 (H28), JF461039 (H32), JF461041 (33), JF461042 (H34), JF461047 (H37) and JF461048 (H42)].


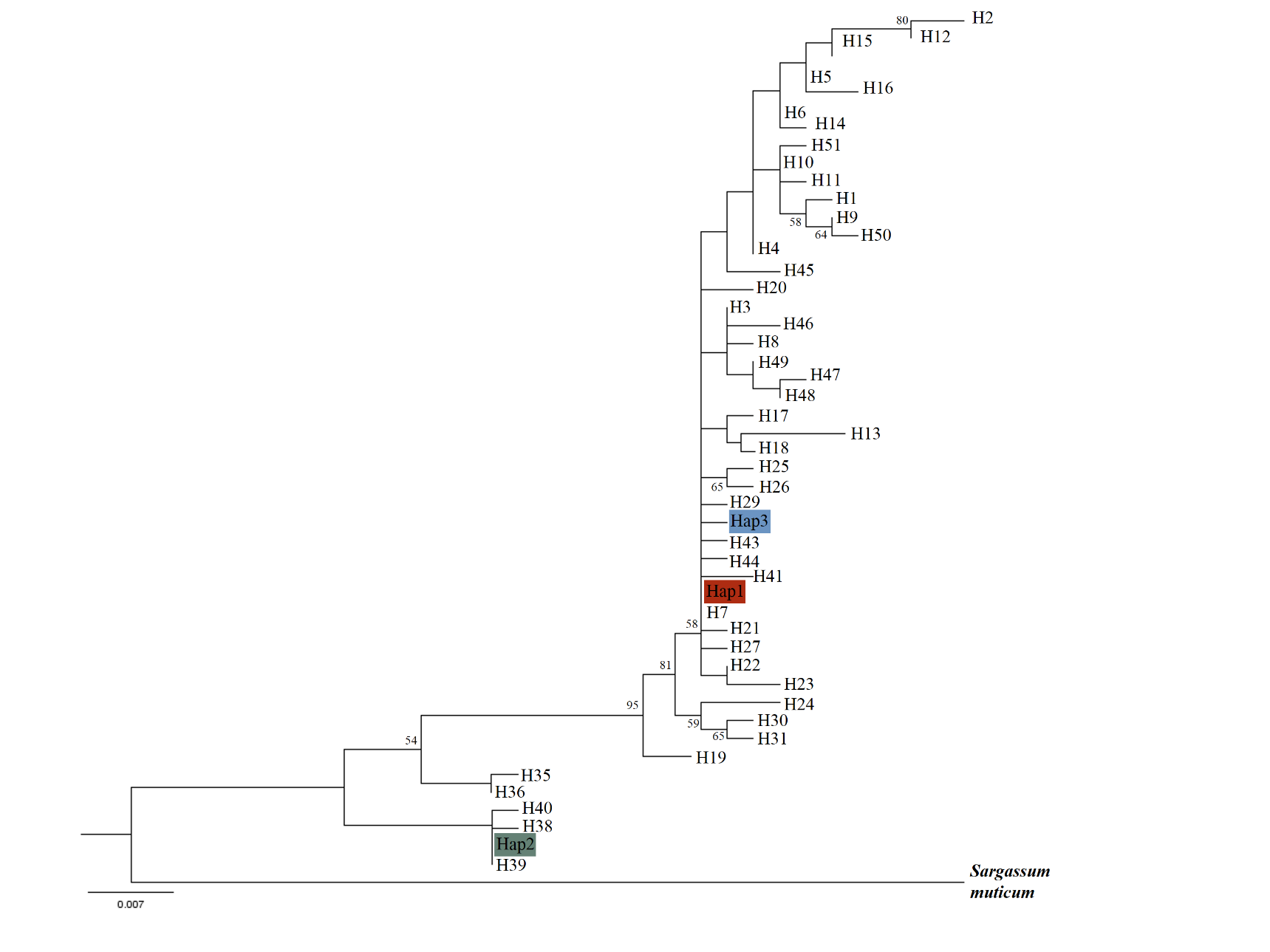

Supplement: Supplementary file 1 — Supplementary Figure S1 [file 41598_2019_44170_MOESM1_ESM.docx]
